# Effects of Resveratrol on Bone Mineral Density in Ovarectomized Rats

**Published:** 2005-06

**Authors:** Qian Lin, Yi-ming Huang, Ben-xi Xiao, Guo-Feng Ren

**Affiliations:** 1 *Department of Nutritional Sciences and Food Hygiene, School of Xiangya Public Health of Central-South University, Hunan, China;*; 2 *Department of Nutrition, Guangdong Provincial People’s Hospital, Guangzhou, China*

**Keywords:** trans-resveratrol, post menopausal osteoporosis, ovarectomized rats, bone mineral density

## Abstract

Hormone replacement therapy (HRT) has been used to prevent osteoporosis in postmenopausal women. However, HRT is not for everyone, due to concerns of side effects as well as increased risk of breast and possibly uterine cancer. Therefore, Dietary alternatives are considered, which include Trans-3,5,4’-Trihydroxystilbene (trans-resveratrol), a phytoestrogen naturally found in grapes, peanuts and wine with beneficial effects in both cardioprotective and chemopreventive. The purpose of this study was to evaluate the effects of trans-resveratrol on the bone metabolism in ovariectomized rats. 48 Rats were assigned to the following groups: sham surgery + normal diet; ovariectomy (Ovx) + normal diet; Ovx + diethylstilbestrol 0.03 mg × kgbw^-1^ × d^-1^;Ovx +Trans-Resveratrol 5 mg × kgbw^-1^ × d^-1^; Ovx + Trans-Resveratrol 15 mg × kgbw^-1^ × d^-1^; Trans-Resveratrol 45 mg × kgbw^-1^ × d^-1^. The rats were fed for 90 days. In the 90th day, OVX + Trans-Resveratrol 45 mg/(kgbw^-1^·d) group had a greater bone mineral density (BMD) than other groups. In the OVX + Trans-Resveratrol 45 mg/(kgbw^-1^·d), indices of endocortical bone formation (ALP 37.90 ± 2.96U/100ml, BGP 1.27 ± 0.10 ng/ml) were greater than those of the other groups, while the index of endocortical bone absorption (TRAP 10.35 ± 1.72 U/L) were lower than those of the other groups. Histopathological examination showed that resveratrol had no endometrial hyperplasia adverse effect. All of these support that resveratroal may have positive effect on postmenopausal osteoporosis prevention.

## INTRODUCTION

Most of Early-stage postmenopausal women suffer from osteoporosis. Postmenopausal osteoporosis (PMOP), shows a rapid decrease in bone mineral density (BMD), which can be due to estrogen deficiency after menopause, is a serious public health problem worldwide (1-3). Estrogen replace therapy (ERT) remains the mainstay for the prevention of postmenopausal, however is challenged by its side effects, particularly the risk of breast and uterine cancers (4, 5). Thus, it is important to find alternatives to estrogen for postmenopausal women to prevent osteoporosis.

Phytoestrogens have many beneficial effects on people’s health. Trans-resveratrol (RES), 3,5,4’-trihydroxylstibene, is a natural compound analogous with the synthetic estrogen diethylstilbestrol and had documented a mixed agonist/antagonist activity for estrogen receptors-α and β (Barry *et al* 1997, Ray *et al* 1999, Jennifer *et al* 2000, Bagchi *et al* 2001) was demonstrated to have beneficial effects on cardiovascular diseases, and may also the osteoporosis in post-menopausal women because of its antioxidant activity (6-9). The purpose of this study was to determine the effects of resveratrol on indices of bone density, and bone formation and resorption in a rat model of aging and menopause. The rats were ovariectomized as a model of peri- and postmenopausal bone loss and replicates many aspects of the changes observed in humans.

## MATERIALS AND METHODS

### Animals & Treatment

Female, healthy adult Sprague-Dawley rats were used in this study (supplied by Laboratory Animal Center, Central south university, Changsha, Hunan, P.R. of China). All of these SD rats were three months of age and 254.91 ± 18.01 gram of weight at the beginning of the study. These animals were kept in a temperature (22 ± 4°C), humidity 60%-65% and light (7:00-19:00) controlled room. Forty-eight SD rats were divided into 6 groups (n=8/group) after twenty-day adaptive feeding period with the standard diet that had calcium content of 1.3%. The rats in five of the groups were ovariectomized and the rats in the other one group were sham operated. The absence of ovarian tissue and plasma estrogen level confirmed the success of the ovariectomy (Ovx). The rats were further divided into the following groups: sham rats fed the base diet (group A), ovariectomized rats fed the base diet (group B), ovariectomized rats with diethylstilbestrol 0.03 mg × kgbw^-1^ × day^-1^(group C), ovariectomized rats with resveratrol 5 mg× kgbw^-1^ × day^-1^(group D), ovariectomized rats with resveratrol 15 mg × kgbw^-1^ × day^-1^(group E) and ovariectomized rats with resveratrol 45 mg × kgbw^-1^ × day^-1^(group F). The rats were fed the diets and tap water and the study continued for 90 days. Food consumption and body weight were measured every week.

### Bone mineral density

BMD was assessed by dual-energy X-ray absorptiometry (DEXA), with the Hologic QDR-4500 A X-ray bone densitometer (Hologic, CO, USA). At the 45th and 90th day after initiated the administration of trans-resveratrol, The total body BMD (T-BMD), as well as the BMD of the second, third, fourth and fifth lumbar vertebrae (mainly cancellous bone) were also scanned and the mean BMD measured.

### Serum estradiol (E2)

Serum estradiol was measured with radioimmunoassay by the XH-6010 RIA counter, (262 factory, Shangxi, P.R. of China). The intra-assay variation was 2.13%, whereas the interassay variation 4.8%.

### Plasma Resveratrol concentration

The resveratrol concentrations were assessed with liquid chromatography assays by LC-6A HPLC (SHIMADZU corporation, Japan). The intra- and interassay variations were both 3.16%.

### Statistical methods

Results were expressed as means ± SD. All data were analyzed with SPSS 11.0 software (SPSS Inc, IL, USA). An ANOVA was first performed to test for any significant differences among groups. When significant, the LSD multiple comparison test were used to determine the specific differences between means. Parametric ANOVA was performed when data were sampled from populations with equal variance. Otherwise, nonparametric methods were selected. Thus, a Kruskal-Wallis test was first performed. If it indicated a significant difference among groups, the Dunnet’s T3 test was used to determine specific differences. To test for any significant differences among days within a group, repeated measures ANOVA was performed and, when significant, the Student-Newman-Keuls multiple comparison test was used to determine the specific differences between means. The level of significance was set at *P*<0.05 for all statistical tests.

## RESULTS

### Body weight gain dynamics

The baseline body weight of the animal groups were similar (*P*>0.05); and all the animal groups had weight gain but not overweighted throughout the experimental period (*P*>0.05). Body weight gain of the estrogen replacement treatment control (C) group was significantly lesser than the other groups (*P*>0.05), and the 45 mg × kg^-1^ × d^-1^ trans-resevestrol (F) group was lesser than the 5 mg × kg^-1^ × d^-1^ (D) and 15 mg × kg^-1^ × d^-1^ (E) and the osteoporosis control (B) groups. The results were listed in Table 1.

**Table 1:** Body weights of rats during the experiment (g, X ± s)

Group	n	Baseline body weight	1th day	90th day	Body weight gain

A (blank control)	8	246.50 ±19.47^a^	254.13 ± 22.44^a^	296.57 ± 28.75^a^	42.44 ± 20.23^ac^
B (osteoporosis control)	8	232.90 ± 15.77^a^	245.00 ± 19.11^a^	293.40 ± 16.30^a^	48.40 ± 19.78^a^
C (estrogen replace control)	8	233.20 ± 16.34^a^	236.20 ± 17.12^a^	259.50 ± 20.13^a^	23.30 ± 19.94^b^
D (5 mg/kg.d)	8	233.40 ± 17.51^a^	242.50 ± 21.08^a^	292.00 ± 19.30^a^	49.50 ± 23.30^a^
E (15 mg/kg.d)	8	234.80 ± 13.38^a^	239.30 ± 24.09^a^	297.56 ± 16.42^a^	52.26 ± 23.42^a^
F (45 mg/kg.d)	8	237.00 ± 14.52^a^	252.70 ± 17.15^a^	287.60 ± 33.89	34.90 ± 25.89^c^

Different superscripts of the iso-column indicate the significant differences between them, *P*<0.05.

### Dynamic estradiol levels

After ovarectomy or shame operation and the twenty-day adaptive feeding period, serum estradiol (E2) levels at the beginning of the experiment, the blank control A group was significantly higher than of all the groups received ovarectomy; and also at the end point of the 90-day experiment (*P*<0.05). It was obvious that estradiol level alterations through the experimental period showed no statistical significance (*P*>0.05), but the normal blank control A and osteoporosis B groups had a little decrement, and the estrogen replacement and the trans-resevarol treatment groups (C, D, E, and F) had a little increment, however. The results were listed in Table 2.

**Table 2:** Serum concentrations of estradiol (pg/mL, X ± s)

Group	N	Serum E2	
		0^th^ day	90^th^ day

A	8	6.52 ± 1.84^a^	6.05 ± 1.31^a^
B	8	3.17 ± 1.27^b^	3.08 ± 0.69^b^
C	8	3.19 ± 1.82^b^	3.38 ± 1.31^b^
D	8	3.14 ± 0.92^b^	3.24 ± 1.18^b^
E	8	2.99 ± 1.05^b^	3.12 ± 0.69^b^
F	8	2.91 ± 0.81^b^	3.28 ± 0.70^b^

### The BMD indices dynamics

**The in vitro whole body and lumbar vertebra BMD dynamics.** Whole body and lumbar vertebral bone specific BMD were assayed during the 45th and 90th day. The BMD parameters of the osteoporosis control (B) group was significantly below the other groups on either whole body or the lumbar vertebra, and either at the 45th or the 90th day of the experiment (*P*<0.05). The estrogen replacement (C) group had BMD parameters higher than that of the osteoporosis (B) group and approached the blank control (A) group (*P*>0.05) except the whole body bone density (*P*<0.05). All the 3 trans-resvesatrol groups approached whole body BMD of the blank control A group (*P*>0.05), but lower lumbar vertebra BMD (*P*<0.05) except the 45 mg × kg^-1^ × d^-1^ F group (*P*>0.05). The actual numerical data of the 3 trans-resvesatrol groups were even higher than that of the estrogen replacement group, but did not have the power to demonstrate statistical significance. The results were listed in Table 3.

**Table 3:** BMDs of the total body and lumbar vertebrae in different periods during the experiment (g/cm^2^, X ± s)

Group	N	BMDs of the total body		BMDs of the lumbar vertebrae	
		45th day	90th day	45th day	90th day

A	8	0.16 ± 0.01^a^	0.17 ± 0.01^a^	0.17 ± 0.00^a^	0.17 ± 0.02^a^
B	8	0.14 ± 0.01^b^	0.15 ± 0.01^b^	0.14 ± 0.01^b^	0.15 ± 0.01^b^
C	8	0.16 ± 0.01^c^	0.16 ± 0.00^c^	0.16 ± 0.01^a^	0.15 ± 0.01^bc^
D	8	0.16 ± 0.00^ac^	0.16 ± 0.00^ac^	0.16 ± 0.01^a^	0.16 ± 0.01^c^
E	8	0.16 ± 0.00^ac^	0.16 ± 0.00^ac^	0.16 ± 0.01^a^	0.16 ± 0.01^c^
F	8	0.16 ± 0.01^ac^	0.16 ± 0.01^ac^	0.16 ± 0.01^a^	0.16 ± 0.01^ac^

**BMD of the isolate lumbar vertebra at end of the 90-day experiment.** At the end point of the 90-day experiment, the lower three lumbar vertebra bones were isolated and had their BMD reassessed. The isolated 4th, 5th, and 6th vertebra BMD of the osteoporosis control B group were lower than that of all the other groups. The 5th lumbar vertebra BMD of the high dose veresatrol (F) group was approximated the blank control (A) group; and all the 6th lumbar vertebrae of the estrogen replacement C and the trans-resvesatrol middle and high dose E, and F groups approximated A group (*P*>0.05) except the low dose 5 mg × kg^-1^ × d^-1^ D group (*P*<0.05). The results were listed in Table 4.

**Table 4:** BMDs of the lumbar vertebrae *in vitro* at the end of the experiment (g/cm^2^, X ± s)

Group	n	Lumbar vertebrae	BMD of L4	BMD of L5	BMD of L6

A	8	0.24 ± 0.03^a^	0.23 ± 0.00^a^	0.24 ± 0.00	0.25 ± 0.00^a^
B	8	0.21 ± 0.01^b^	0.21 ± 0.01^bc^	0.21 ± 0.01^b^	0.22 ± 0.01^b^
C	8	0.23 ± 0.01^a^	0.22 ± 0.01^c^	0.23 ± 0.01^ac^	0.24 ± 0.01^ac^
D	8	0.23 ± 0.00^a^	0.22 ± 0.01^ac^	0.22 ± 0.01^bc^	0.23 ± 0.01^c^
E	8	0.23 ± 0.01^a^	0.22 ± 0.00^bc^	0.22 ± 0.01^ab^	0.24 ± 0.02^ac^
F	8	0.24 ± 0.01^a^	0.22 ± 0.01^ac^	0.23 ± 0.02^ac^	0.25 ± 0.01^ac^

**BMD of the femur and tibia bones evaluated as a whole and in the 7 femur sensitive regions of interest (FROI).** The whole femur and tibia bones BMD of group A was significantly higher than all the other groups (*P*>0.05) except the 45 mg × kg^-1^ × d^-1^ high dose trans-resvesatrol group F (*P*>0.005). When the femur and tibia bones were evaluated in 7 artificial divided sensitive regions from the proximal terminal to the distal, all the TROI parameters of the group B were the lowest (*P*<0.05). Both the FROI-1 and -2 of the B, C, D, and E groups were significantly lower than that of the A group (*P*<0.05), whereas the 45 mg × kg^-1^ × d^-1^ high dose trans-resvesatrol group approximated the A group (*P*>0.05). The BMDs of A and F group at both the FROI-6 and -7 showed no significant difference (*P*>0.05), and all the three trans-resvesatrol groups had higher BMD density parameters than that of the osteoporosis control group B, but only the group F in FORI-6 and group E and F had the power to demonstrate statistical significance (*P*<0.05). The results were listed in Table 5 and 6.

**Table 5:** BMDs of the femur and the femur sensitive regions of interest (g/cm^2^, X ± s)

Group	n	BMD of the femur	FROI-1	FROI-2	FROI-3	FROI-4	FROI-5	FROI-6	FROI-7

A	8	0.25 ± 0.02^ac^	0.32 ± 0.02^a^	0.21 ± 0.02^a^	0.20 ± 0.02^a^	0.22 ± 0.02^a^	0.24 ± 0.02^a^	0.26 ± 0.02^a^	0.25 ± 0.01^a^
B	8	0.22 ± 0.00^b^	0.29 ± 0.01^b^	0.18 ± 0.01^b^	0.19 ± 0.01^ab^	0.21 ± 0.01^bc^	0.22 ± 0.01^b^	0.24 ± 0.01^b^	0.23 ± 0.01^b^
C	8	0.23 ± 0.02^bd^	0.30 ± 0.02^b^	0.19 ± 0.02^b^	0.18 ± 0.01^b^	0.20 ± 0.02^c^	0.22 ± 0.02^b^	0.25 ± 0.01^bc^	0.24 ± 0.01^bc^
D	8	0.23 ± 0.00^bd^	0.30 ± 0.01^bc^	0.19 ± 0.01^bc^	0.20 ± 0.01^a^	0.22 ± 0.01^a^	0.23 ± 0.01^ac^	0.25 ± 0.00^ab^	0.23 ± 0.01^bc^
E	8	0.24 ± 0.01^d^	0.30 ± 0.02^c^	0.19 ± 0.01^c^	0.20 ± 0.01^a^	0.22 ± 0.01^ab^	0.22 ± 0.01^bc^	0.25 ± 0.01^ab^	0.24 ± 0.01^c^
F	8	0.24 ± 0.01^d^	0.31 ± 0.02^ac^	0.20 ± 0.02^ac^	0.20 ± 0.01^a^	0.22 ± 0.01^a^	0.23 ± 0.02^ac^	0.26 ± 0.02^ac^	0.24 ± 0.01^ac^

**Table 6:** BMDs of the tibia and the tibia sensitive regions of interest (g/cm^2^, X ± s)

Group	n	Tibia as a whole	TROI-1	TROI-2	TROI-3	TROI-4	TROI-5	TROI-6	TROI-7

A	8	0.22 ± 0.01^a^	0.30 ± 0.02^a^	0.21 ± 0.01^a^	0.21 ± 0.04^a^	0.19 ± 0.01^ac^	0.20 ± 0.01^a^	0.19 ± 0.01^ac^	0.22 ± 0.01^a^
B	8	0.20 ± 0.01^b^	0.26 ± 0.01^b^	0.19 ± 0.01^b^	0.18 ± 0.01^bc^	0.18 ± 0.01^ab^	0.189 ± 0.01^bc^	0.18 ± 0.01^ab^	0.21 ± 0.01^ab^
C	8	0.21 ± 0.02^bc^	0.28 ± 0.02^c^	0.19 ± 0.01^b^	0.18 ± 0.01^bc^	0.17 ± 0.01^b^	0.18 ± 0.01^b^	0.18 ± 0.01^b^	0.20 ± 0.01^b^
D	8	0.21 ± 0.01^bc^	0.28 ± 0.01^c^	0.20 ± 0.01^ab^	0.19 ± 0.01^bc^	0.19 ± 0.01^ac^	0.19 ± 0.01^ac^	0.19 ± 0.01^bc^	0.21 ± 0.01^ab^
E	8	0.21 ± 0.01^ab^	0.28 ± 0.01^c^	0.20 ± 0.01^ab^	0.19 ± 0.01^ac^	0.18 ± 0.01^ac^	0.19 ± 0.01^ac^	0.20 ± 0.01^ac^	0.21 ± 0.01^ab^
F	8	0.22 ± 0.01^ac^	0.28 ± 0.01^c^	0.20 ± 0.02^ab^	0.19 ± 0.01^ac^	0.20 ± 0.01^c^	0.20 ± 0.01^a^	0.20 ± 0.01^c^	0.21 ± 0.01^ab^

## DISCUSSIONS

Trans-Resveratrol (resveratrol) has been shown in several studies to significantly modulate biomarkers of bone metabolism. Mizutani *et al* found that trans-resveratrol may promote bone formation by enhancing the osteoblasts activity and stimulate osteoblasts proliferation and differentiation (10, 11). But, there is no direct evidence supporting its inhibitory effect towards bone loss. Therefore, in present study, the effects of trans-resveratrol on bone mineral density (BMD) and bone metabolism indices were examined in the ovariectomized (OVX) rat model.

The present experiment used small, median, and high dose trans-resveratrol groups in comparison with the estrogen replacement control to evaluate the effect of trans-resveratrol on BMD of the rat PMOP model. The results demonstrated that trans-resveratrol do provide protective effect on ovarectomized rat BMD. The 3 different trans-resveratrol dose animals lumbar vertebrae BMD was higher than that of the control model group and approximated the normal blank control group during the 45th day, at the middle of the experiment; at the end point, the 90th day, of this experiment, the trans-resveratrol groups still exhibited positive effect on BMP, the whole body BMD remained at the level of normal controls. In addition, as to the protective effect on lumbar vertebra, the 45 mg × kg^-1^ × d^-1^ trans-resveratrol group approximated the normal control group level, and was surpassed the 0.03 mg × kg^-1^ × d^-1^ ethylestradiol group.

Since the trabecular bone density decrement is more sensitive to hypo-estrogenemia, the lumbar vertebra composed mainly trabecular bone and the physis of long bones were of most important consideration in PMOP bone lose. At the end point of this experiment, ethylestradiol and the 3 trans-resveratrol groups showed significant protection on ovarectomy induced BMP losses, which was approximated the normal controls. As to the individual lumbar vertebra, the protective effect was progressively increasing caudally, with the best results on the 6th, followed by the 5th vertebra, which were reached statistical significance of *P*>0.05; except the 4th vertebra, which may suggest a site specificity. The protective effect of trans-resveratrol on femur was better than tibia as a whole. At the proximal end of tibia, the TROI-1, all -3 in trans-resveratrol groups demonstrated significant protective effect; and the same were demonstrated on FROI-1, -2, -6, and -7 of the femur by both the 15 and 45 mg×kg^-1^×d^-1^ trans-resveratrol groups. In summary, the phytoestrogen analogous substance trans-resveratrol demonstrated protective effect on BMD lose in ovarectomized rat model, as trans-resveratrol effectively preserved the BMD; and the 45 mg × kg^-1^ × d^-1^ dose group demonstrated the best protective effect, which might suggests a dose-dependent manner, although the experimental design did not have the power to demonstrate a statistical significance in all of the experimental parameters.

Studies reported the enhanced expression of cytokines such as TGFs and eNOS by trans-resveratrol, which had effects on skeletal tissue quality (12, 13). And also studies reported significant inhibitive effects of trans-resveratrol on the expression and/or production of cytokines such as IL-1, IL-6, TNF-α, and PGE2, which enhance the osteoclasts activities and resultant in reduced bone resorption on the background of estrogen deficiency (8, 9, 14, 15).

In conclusion, the present study demonstrated that a daily resvertrol intake in adult ovariectomized rats reduced bone turnover and also reverse a previous bone loss. Furthermore, it appeared that the highest 45 mg × kg^-1^ × d^-1^ resveratrol administration levels were more effective in depressing the ovariectomy-induced increase in bone turnover (and in bone resorption specifically) than other dose. Therefore, ingestion levels of resveratrol should be considered to improve bone health in a preventive rather than a curative approach of human postmenopausal osteoporosis.
